# Tendon Adaptations to Eccentric Exercise and the Implications for Older Adults

**DOI:** 10.3390/jfmk4030060

**Published:** 2019-08-20

**Authors:** Jonathan I Quinlan, Marco V Narici, Neil D Reeves, Martino V Franchi

**Affiliations:** 1 School of Sport, Exercise and Rehabilitation Sciences, University of Birmingham, Birmingham B15 2TT, UK; 2 NIHR Birmingham Biomedical Research Centre, University Hospitals Birmingham, NHS Foundation Trust and University of Birmingham, Birmingham B15 2GW, UK; 3 Department of Biomedical Sciences, Institute of Physiology, University of Padua, 35131 Padua, Italy; 4 Department of Life Sciences, Research Centre for Musculoskeletal Science and Sports Medicine, Manchester Metropolitan University, Manchester M1 5GD, UK

**Keywords:** tendon, ageing, eccentric, resistance training, eccentric training

## Abstract

The purpose of this short review is to discuss the effects of eccentric exercise in modifying the properties of tendon tissue in healthy individuals. The tendon provides a mechanical link between muscle and bone, allowing force transmission to the skeleton, and thus, its properties have significant functional implications. Chronic resistance training has long been shown to increase the stiffness and Young’s modulus of the tendon and even tendon cross-sectional area. However, as the tendon responds to the amount and/or frequency of strain, it has been previously suggested that eccentric training may result in greater adaptations due to the potential for greater training loads. Thus, this review discusses the effects of eccentric training upon healthy tendon tissue and compares these to other training modalities. Furthermore, it has been reported that the tendon may undergo adverse age-related changes. Thus, this review also discusses the potential application of eccentric resistance training as a preferential modality for counteracting these age-related changes. We conclude that while there may be no difference between contraction types for overall tendon adaptation, the lower demands of eccentric contractions may make it more appealing for the elderly population.

## 1. Introduction

**Figure 1:**
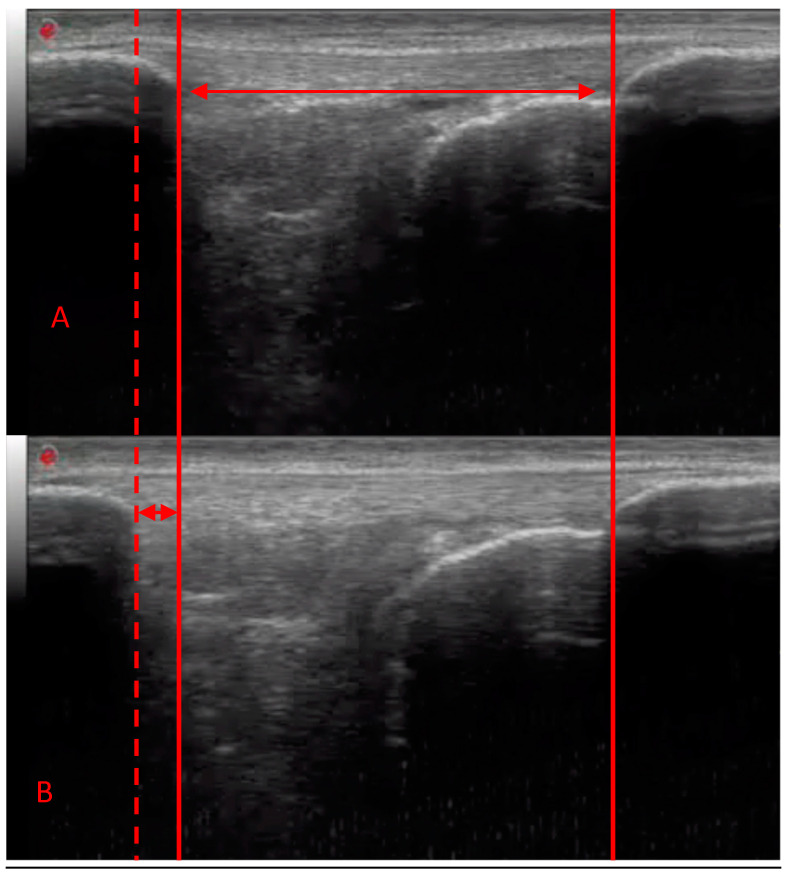
Ultrasonography images of the patellar tendon obtained at rest (**A**) and during a maximal isometric ramped contraction (**B**). The solid lines demonstrate the resting tendon length as measured between the apex of the patella and the tibial tuberosity. The dashed line represents the elongation of the tendon that has occurred during contraction.

Skeletal muscle has long been the focus of research into loading adaptations, inherently due to its functional and metabolic importance. It is highly adaptable to a large variety of exercise stimuli, including resistance exercise training (RET). Conventional RET typically involves a movement where an external load is lifted and consequently lowered under control. Therefore, conventional RET contains both a shortening (concentric) and lengthening (eccentric) contraction in the same movement. Due to the differing physiological nature of these contractions, optimising each of these muscle contraction types has generated considerable research interest [1]. However, whilst it is the muscle that is responsible for generating force, it is the role of the tendon to transmit this force to the bone, allowing movement and locomotion. Thus, the tendon is an integral part of the appropriately named muscle–tendon unit. Historically, tendon tissue has been considered an inert tissue. However, over the last ~15 years, it has been shown to be metabolically active and respond to mechanical loading by modification of its mechanical properties, albeit how active still appears contentious [2,3]. Nonetheless, the adaptive ability of tendon mechanical properties with exposure to increased strain through RET is well accepted [4,5,6,7]. Typically, measures of tendon mechanical properties include stiffness and Young’s modulus. In humans, these variables are obtained during an isometric ramped contraction whilst utilising dynamometry, ultrasonography, electromyography and goniometry (for the method, see [8]). Tendon stiffness is calculated by dividing the change in force by the change in elongation of the tendon, i.e., how much force is required to elongate the tendon by a given amount (Figure 1).

Young’s modulus, on the other hand, is calculated by dividing tendon stress by tendon strain, thus taking into consideration the dimensions of the tendon, i.e., original length and cross-sectional area (CSA). Whilst many studies show increases in the mechanical properties following RET, the same cannot be said for tendon hypertrophy. In many cases, tendon hypertrophy does not occur following RET; however, some studies have shown hypertrophy may occur mainly towards the osseous–tendinous junctions (OTJ) [9]. It is suggested that the compressive loads that occur at the OTJ, which are absent in the mid portion of the tendon, may be responsible for this regional hypertrophy. Others have shown that long-term, or lifelong exercise is associated with a larger tendon CSA [10,11]. It is likely that in this case, there has been sufficient loading over a prolonged period of time which has resulted in hypertrophy. For more comprehensive reviews of tendon adaptation to exercise, see [12,13,14]. In contrast to previous reviews, the purpose of this brief review is to focus on the comparison of eccentric and concentric contractions in improving tendon properties in young individuals. Additionally, we discuss the application of eccentric exercise in counteracting age-related declines in tendon properties and function.

## 2. Eccentric Exercise and Tendon Adaptation

The resident fibroblasts of tendon tissue, tenocytes, respond to strain by altering collagen expression, increasing collagen synthesis and triggering the remodelling process. Notably, it has been shown that the degree of tendon adaptation is dependent on the magnitude of strain, such that greater strains induce greater acute responses [15,16]. Previous research has compared high- and low-intensity RET, whereby it was found that higher training loads and, hence, higher levels of strain resulted in positive changes to tendon mechanical properties, while low-intensity RET did not [6]. The same conclusion was found even when the total training volume was controlled for, reiterating that the magnitude of acute load is a significant factor for tendon adaptation [9]. With this in mind, the physiological basis of eccentric contractions facilitates higher force production, and hence, utilisation of larger training loads [1]. Therefore, it is logical to suggest that these higher training loads would provide the potential for greater tendon strain and thus lead to either greater or faster tendon adaptation than either concentric or conventional RET. As such, it is no surprise that this form of exercise has regularly been used as a method to treat tendinopathies [17,18]. However, the treatment of tendinopathies and other tendon pathologies remains a complicated topic. While tendinopathies are typically associated with changes in tendon size, tendon compliance, collagen fibrillar patterns and altered vascularisation [19] these changes can occur without symptoms, pain or altered function. Nonetheless, the treatment of tendinopathy via eccentric RET falls out of the scope of this review; for more detail on this topic, please see [19].

Eccentric training has a major role in injury prevention and improving tendon properties in healthy individuals. Several studies have investigated eccentric training of the triceps surae and Achilles tendon; however, the results are somewhat conflicting. One study completed in 2008 employed 6 weeks of eccentric heel drop training in young individuals [20], according to the Alfredson protocol [21]. Mahieu and colleagues found that while there were positive changes in ankle dorsiflexion and decreases in passive resistive torque, there was no change in Achilles tendon stiffness. Meanwhile, another study which employed 6 weeks of eccentric heel drop training (also Alfredson protocol) found that Achilles tendon stiffness instead became decreased [22]. However, in contrast to these two studies that showed no chance or a decrease, Achilles tendon stiffness has been shown to increase following 7 weeks of unilateral high-intensity eccentric RET in young males [23]. Similarly, another study which utilised 12 weeks of eccentric triceps surae RET also showed a significant increase in Achilles stiffness and Young’s modulus [24]. Interestingly, this study also investigated the time course of Achilles adaptation, in which they found significant increases in biomechanical properties after only 4 weeks of eccentric RET. In conclusion of these studies, it has been shown that Achilles tendon stiffness may increase, decrease or remain unaltered with eccentric training. However, as previously mentioned, changes in tendon biomechanical properties require sufficient loading such that a threshold must be surpassed [13]. Thus, it is likely that these conflicting results spawned from differences in loading protocol. The studies which demonstrated a decrease or no change in Achilles tendon stiffness utilised the Alfredson protocol. The Alfredson model, which is typically used to treat tendinopathy, consists of body weight eccentric heel drop exercises. Thus, the only loading that occurs with this protocol is induced via an individual’s body weight. By contrast, those which demonstrated increases in Achilles tendon stiffness utilised high-intensity eccentric resistance training. One study implemented 6 sets of 6 reps at 120% of concentric 1 repetition max (1RM) [23], and the other initially completed 3 sets of 10, progressing to 5 sets of 10, of maximal eccentric contractions at 30°/s [24]. Therefore, the loading that occurred in these two studies compared to the Alfredson protocol would have been substantially larger. It is likely that while the Alfredson protocol may have large clinical benefit [17], the loading is insufficient to alter tendon stiffness and/or Young’s modulus in healthy individuals.

## 3. Are eccentric Contractions More Effective than Concentric for Tendon Adaptation?

While it has been shown that eccentric exercise is capable of inducing positive changes in various tendon properties, very few have directly compared eccentric exercise interventions to either concentric or conventional training. Initial animal models provided useful, albeit inconclusive, information in regard to this topic. Early work by Heinemeier provided young adult Sprague–Dawley rats with 4 days of concentric, eccentric or isometric training of the gastrocnemius and in-series Achilles tendon via stimulation of the sciatic nerve under anaesthetic [25,26]. Twenty-four hours after the final training bout, the Achilles tendon was harvested: The main findings showed that many key regulators of tendon remodelling were upregulated following exercise, such as collagen type 1, collagen type 3, transforming growth factor-β-1 (TGF-β-1), insulin growth factor-1Ea (IGF-1Ea), mechano growth factor (MGF), matrix metalloproteinase (MMP)-2, tissue inhibitor of MMP (TIMP) 1 and 2 and lysyl oxidase (LOX). These results were independent of contraction type; despite a significantly higher force–time integral in eccentric training compared to concentric and isometric. Nonetheless, there were no measures of any biomechanical properties; thus, the above results assume that similar changes in RNA expression between concentric and eccentric training would evoke equivalent changes in biomechanical properties. By contrast, a more recent rat model investigated the effect of 5 weeks of uphill (concentric) or downhill (eccentric) treadmill running on biomechanical properties of the tricipital, patellar and Achilles tendons [27]. After completion of the exercise intervention, the biomechanical properties were assessed via an in vitro displacement-rate controlled tensile force to induce rupture assessment. The extracted tendon was clamped and elongated at a rate of 1mm/s until rupture occurred. Following rupture, the forced which induced rupture was recorded. The authors found that there was a significant increase in rupture force in both the patellar and tricipital tendons in the eccentric group compared to their untrained group, but not in the concentric group compared to controls, indicating a greater tendon adaptation through eccentric exercise.

**Figure 2:**
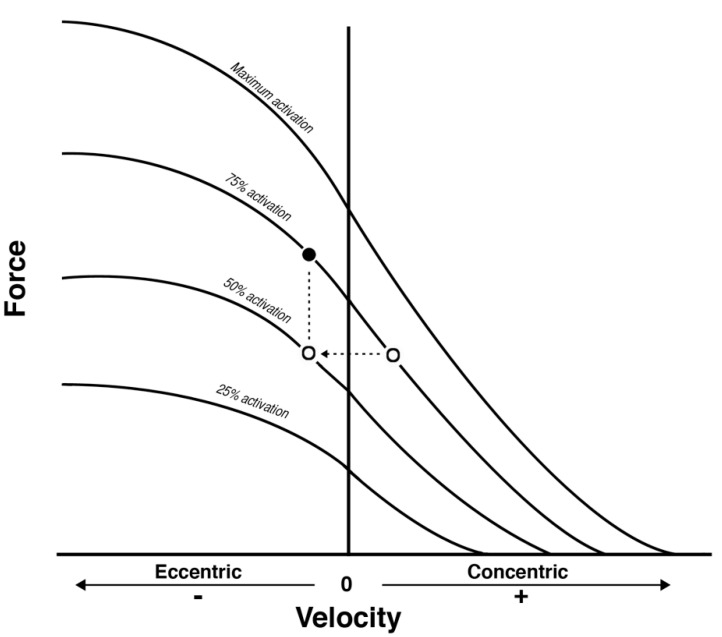
Schematic representation of force–velocity curves corresponding to different levels of muscle activation. The open circles demonstrate the theoretical scenario during a conventional movement, such that the eccentric contraction would belong to a different force velocity curve which is at lower activation, indicated by the dashed arrow. Whereas the closed circle represents an eccentric contraction completed with the same level of activation as the concentric contraction, whereby force production is notably higher. The dashed line represents the theoretical difference in force produced between eccentric contractions. (Reprinted with permission from Reeves et al., 2009 [29]).

The animal models appear inconclusive, as some show advantages for eccentric while others show no difference [25,26,27]. These differences are perhaps due to methodological differences, i.e., loading, timescales and the method of assessment. However, as with any animal model, it is often a challenge to transfer these conclusions to humans. Unfortunately, only a select few studies have directly compared the effects of long-term concentric or eccentric exercise interventions on various tendon properties. One study completed by Malliaras and colleagues compared concentric (80% concentric 1RM) standard load eccentric (80% concentric 1RM) and high load eccentric (80% eccentric 1RM) RET in a 12-week intervention within a healthy young male cohort [7]. It was found that patellar tendon stiffness and modulus increased in all training groups, with no differences between groups when obtained over the 50–75% torque interval. However, when modulus was obtained from the 75–100% force interval, only the high load eccentric group demonstrated an increase from baseline. The authors suggested that high load eccentric training may have greater influences on tendon properties at higher torque levels. Significantly, there were no changes in tendon CSA, and thus, it is likely that the changes in modulus observed were induced by changes in the material properties of the tendon. It is important to highlight that while the training was formed of either concentric or eccentric contractions, the authors utilised a pragmatic approach in which the weight would be returned to the ‘starting position’ in bilateral fashion. Therefore, the training programme was not strictly pure in terms of contraction mode, as training did include both concentric and eccentric contractions. A more recent study completed by Kubo and colleagues compared changes in both stiffness and blood circulation in the patellar tendon of heathy young males in response to 12 weeks of either 80% 1RM concentric or eccentric RET [28]. In contrast to the aforementioned Malliaras study, Kubo demonstrated that patellar tendon stiffness only increased in the concentric group and not in the eccentric group [28]. While these results appear conflicting, there are several possible explanations as to why these studies contradict one another. Kubo utilised identical training loads for concentric and eccentric contraction, which is in contrast to Malliaras, who also utilised a high load eccentric group, i.e., one which trained at 80% of eccentric 1RM. Consequently, when utilising the same absolute load for both contractions, there would be a lower recruitment of motor units during the eccentric contraction phase. In turn, this would result in the eccentric contraction being on a different force–velocity curve with a lower level of activation (Figure 2) [29]. Thus, in the Kubo study, the eccentric group would have been relatively underloaded in comparison to the concentric group (and therefore not using the eccentric contraction phase to its full potential). The other significant difference between the two is the force intervals at which tendon stiffness/modulus was obtained. While Malliaras compared over multiple force intervals (50–75% and 75–100%), it appears that the measurements obtained by Kubo were done so over the maximal 50% of force (i.e., 50–100% force interval). As previously mentioned, it is possible that high-intensity eccentric RET has greater influences on biomechanical properties at higher force levels; thus, an effect of ECC RET may have been missed. A third study, while not investigating tendon biomechanical properties, compared the effect of 12 weeks of concentric or eccentric RET in combination with whey protein supplementation on patellar tendon CSA [30]. Despite measuring a different variable, the same conclusion was reached. The authors compared the change in proximal tendon CSA and found that both concentric and eccentric RET induced significant increases from baseline; however, these values were similar in both training modalities. Thus, it appears that at least in young males, the tendon may adapt similarly to concentric and eccentric training in terms of mechanical properties and hypertrophy. Nonetheless, to truly ascertain whether this is the case, an appropriately loaded and isolated eccentric contraction must be used. Finally, when comparing concentric and eccentric contractions, it is worth noting that the overwhelming majority of research is carried out on lower limb tendons (patellar and Achilles). This apparent bias may be due to their specific role within energy retention and power amplification during movement [31,32]. Thus, both the patellar and Achilles tendon have high functional significance and, hence, high research interest. Nonetheless, results may be translated to the upper extremities, with the caveat that tendon dimensions and, hence, tendon properties vary due to anatomical location [33]. It is possible that a tendon of similar size and function would respond in similar fashion to either the patellar or Achilles tendon.

## 4. Rationale of Using Eccentric Exercise for the Elderly Tendon

It is well established that skeletal muscle undergoes many negative age-related changes, including a reduction in strength, reduced muscle mass and a consequent reduction in functional capacity. However, what is not as well documented is the age-related changes in tendon tissue. Although not a universal finding, in humans, the tendon has been shown to undergo an age-related reduction in the biomechanical properties, such as stiffness and modulus [8,10,34]. However, it is still a point of debate as to whether the tendon tissue itself deteriorates with ageing such that the tendon becomes more compliant, or instead whether these changes are due to a reduction in loading typically seen in ageing [14]. Nonetheless, these biomechanical alterations can have implications on key performance characteristics, such as the rate of force development [8,35], which is also known to decrease with ageing [36,37].

The aged tendon has been shown to retain its ability to respond to increases in mechanical loading such as that experienced with exercise [6,38]. Unfortunately, the majority of studies which have investigated tendon adaptation to exercise in the elderly utilised conventional resistance exercise. Therefore, it is relatively unknown how effective eccentric exercise is in the elderly in terms of inducing positive tendon changes, and further, whether these adaptations are superior to concentric or conventional training. One would assume that based on the data shown in the young, a similar story exists in the elderly, i.e., the tendon is insensitive to the mode of contraction and simply responds to sufficient levels of mechanical loading. If it is the case that there is no difference between concentric or conventional and eccentric only RET, it would be tempting to suggest that eccentric may have no additional benefit for the elderly tendon over resistance training of a given load regardless of contraction type. However, there are many additional characteristics of eccentric RET which make it a more appealing modality for the elderly population. Eccentric contractions have been shown to induce greater oscillations and vibrations within the tendon tissue in comparison to those seen in concentric contractions [39,40,41]. It has been suggested that these oscillations have been implicated with therapeutic effects on the tendon and, thus, may be particularly beneficial for an elderly population. Nonetheless, perhaps one of the greatest benefits of eccentric contractions is owed to their physiological nature, as they are significantly less metabolically demanding than concentric or isometric contractions [42,43]. It is known that there are many mitochondrial alterations with ageing, with inherently reduce the metabolic capacity of skeletal muscle [44,45]. As such, it is no surprise that eccentric exercise has often been touted as an attractive training modality for chronically ill populations [46,47]. Similarly, eccentric contractions would allow older individuals to train at higher intensity levels, without placing additional stress onto a compromised mitochondrial system. Notably, eccentric strength has been shown to be maintained in the elderly [48] and so provides further rationale to utilising this form of exercise. Finally, eccentric exercise has been shown to induce a lower rating of perceived exertion compared to conventional training in older individuals, despite utilising significantly higher training loads [29]. Nonetheless, one concern with utilising high load eccentric RET in an older population is the potential for muscle damage and/or tendon injury in an already weakened muscle–tendon unit. However, it has been shown that following the first bout of eccentric RET, the risk of muscle damage, even in older adults, is severely reduced, a phenomenon known as the repeated bout effect [49,50]. Nevertheless, if there are additional concerns in utilising ECC RET, due to previous injuries or otherwise, it is recommended the intensity, volume and frequency be adjusted appropriately on an individual basis [51].

## 5. Conclusions and Future Directions

In light of this brief review, it is clear that eccentric RET has potential beneficial implications for tendon tissue. However, what is not as conclusive is whether the adaptations seen with eccentric contractions are greater than that of concentric or conventional training. This lack of definitive results likely stems from differences in study protocols, such as the combination of contraction type and under loaded eccentric contractions. Nonetheless, it is possible that the greater levels of strain imposed during eccentric contractions may induce a faster adaptation of the tendon. However, the time-course of both concentric and eccentric contractions have yet to be compared. Thus, it is plausible that while the overall magnitude of adaptation is similar, the speed with which this change occurs may differ between the two. Further, there is a need to investigate the potential benefits of eccentric RET in the aged tendon. As mentioned, negative changes in the tendon occur with natural ageing, which can result in functional decline. Eccentric RET offers a particularly appealing training modality for the older population due to its lower metabolic cost and lower perceived effort while utilising greater training loads. However, the full potential of eccentric RET in inducing positive tendon adaptation in the elderly does not seem to have been fully appreciated.
